# Case Report: Contiguous presentation of anti-MDA5 juvenile dermatomyositis and anti-AQP4 neuromyelitis optica spectrum disorder in an adolescent patient

**DOI:** 10.3389/fped.2024.1376088

**Published:** 2024-06-14

**Authors:** Rebecca E. Wiersma, Zachary R. Shaheen, Colleen K. Correll, Patricia M. Hobday

**Affiliations:** ^1^ Department of Pediatrics, University of Minnesota Medical School, Minneapolis, MN, United States; ^2^ Division of Rheumatology, Allergy & Immunology, Department of Pediatrics, University of Minnesota Medical School, Minneapolis, MN, United States

**Keywords:** juvenile dermatomyositis, dermatomyositis, neuromyelitis optica spectrum disorder, anti-melanoma differentiation-associated proteins 5, anti-aquaporin-4, case report

## Abstract

Neuromyelitis optica spectrum disorder (NMOSD) is a rare inflammatory disorder of the central nervous system (CNS) that is known to be associated with other neurologic and organ-specific autoimmune conditions. There has been increasing recognition of the association between NMOSD and systemic autoimmune disease, most commonly systemic lupus erythematosus and Sjogren's syndrome. We report a case of an adolescent presenting with anti-melanoma differentiation-associated protein 5 juvenile dermatomyositis (anti-MDA5 JDM) and NMOSD, exhibiting clinical features of myelitis, polyarthritis, myositis, and skin involvement. Currently, only two other published cases have described NMOSD associated with anti-MDA5 dermatomyositis, both in adults. To the best of our knowledge, this is the first reported case in an adolescent patient.

## Introduction

Neuromyelitis optica spectrum disorder (NMOSD) is a rare inflammatory disorder of the central nervous system (CNS) characterized by immune-mediated demyelination, predominantly affecting the optic nerves and the spinal cord. Its estimated overall incidence in children and adults ranges from 0.5 to 4 per 100,000 (1). Pediatric-onset NMOSD is estimated to account for 3%–5% of cases (1). NMOSD classically presents with vision loss (optic neuritis) and sensory and motor deficits (transverse myelitis). It is often relapsing and has only been formally recognized as a disease entity separate from multiple sclerosis (MS) in 2004 (2). NMOSD is associated with antibodies to the aquaporin-4 water channel (anti-AQP4). According to the 2015 NMOSD consensus diagnostic criteria, diagnosis can be made with anti-AQP4, the presence of at least one core clinical characteristic (including optic neuritis, acute myelitis, or area postrema syndrome), and exclusion of alternate diagnoses (3). Acute treatment comprises plasma exchange transfusion (PLEX) and high-dose IV steroids, and rituximab is used as maintenance treatment (4).

NMOSD has been associated with other neurologic and organ-specific autoimmune conditions, most commonly myasthenia gravis (MG) and autoimmune thyroid disease (5). Recently, there has also been increasing recognition of the association between NMOSD and rheumatic diseases. Research to date has primarily focused on the association with systemic lupus erythematosus (SLE) and Sjogren's syndrome, although recent case reports have begun to reveal associations with additional systemic autoimmune disease processes (5–9).

One such autoimmune disease is juvenile dermatomyositis (JDM). JDM is a rare inflammatory myopathy with an estimated incidence of 2–4 cases per 1 million children (10). Characteristic symptoms include skin rash (heliotrope rash, malar rash, and Gottron papules), proximal muscle weakness, and myositis. JDM is associated with myositis-specific autoantibodies (11). Anti-melanoma differentiation-associated protein 5 (anti-MDA5) is a myositis-specific autoantibody that is associated with a high mortality risk typically due to rapidly progressive interstitial lung disease (ILD) (11). To date, only two other published cases have described NMOSD occurring near-simultaneously with dermatomyositis and MDA-5 antibody positivity. Notably, both cases were in older adults (12, 13). Here, we report the first known adolescent patient with a near-simultaneous onset of JDM and NMOSD.

## Case description

**Figure 1:**
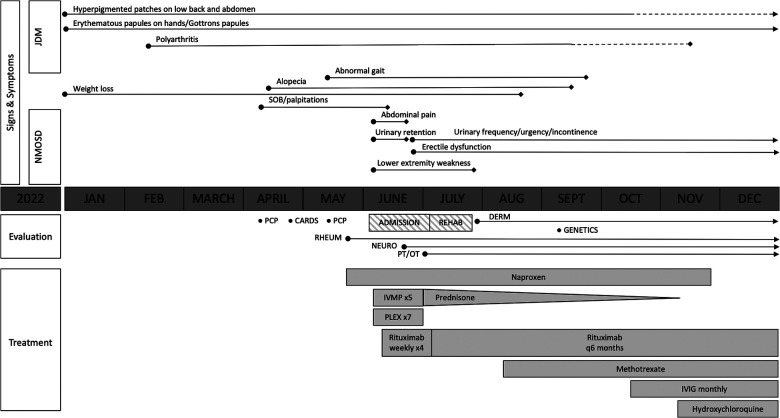
Overview of the patient's clinical course. The solid lines represent the presence of symptoms, with the length of the lines indicating symptom duration. The dotted lines represent a partial alleviation of symptoms. Transverse myelitis due to neuromyelitis optica spectrum disorder (NMOSD) improved following the initiation of intravenous methylprednisolone (IVMP), plasma exchange (PLEX), and rituximab, although the patient has had ongoing urinary frequency and erectile dysfunction. Juvenile dermatomyositis (JDM)-related polyarthritis improved with the addition of methotrexate; however, he had persistent cutaneous disease prompting the addition of intravenous immunoglobulin (IVIG) and hydroxychloroquine.

Approximately 5 months prior to presentation, a 17-year-old Vietnamese male developed dry patches on his hands, lower back, and abdomen, which was initially treated as possible atopic dermatitis (clinical course summarized in Figure 1). He also experienced 3 months of chronic progressive joint symptoms characterized by pain and morning stiffness of his knees, elbows, and fingers that eventually precluded him from being able to efficiently complete schoolwork or perform as a training pianist. Lab workup was notable for pancytopenia [white blood count (WBC), 3.3 × 10^3^/µl; hemoglobin, 12.3 g/dl; platelet count, 135 × 10^3^/µλ], hyperferritinemia (855 ng/ml with a reference range of 22.0–275.0 ng/ml), and aspartate transferase (AST) of 45 IU/L (reference range, 3–39 IU/L). The complete metabolic panel (CMP) was otherwise normal, and he had a normal peripheral smear, urinalysis (UA), thyroid-stimulating hormone (TSH), and negative anti-HIV1/2 antibody and hepatitis C antibody. He was referred to pediatric rheumatology and hematology/oncology for further workup.

**Figure 2:**
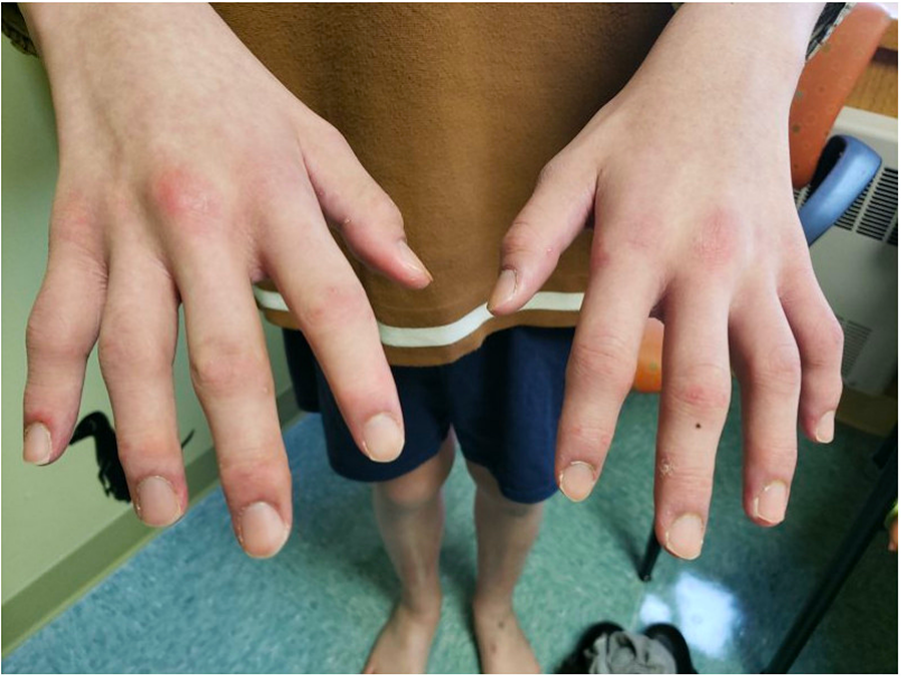
Physical exam findings at the time of initial evaluation by pediatric rheumatology. Erythema was seen overlying multiple knuckles and the distal fingertips without associated papules or nodules. There was polyarthritis of the metacarpophalangeal (MCP), proximal interphalangeal (PIP), and distal interphalangeal (DIP) joints bilaterally. He would eventually develop a more classic pattern of Gottron papules (erythema over MCPs with associated papular eruption).

On initial presentation to pediatric rheumatology, his exam was notable for symmetric polyarthritis affecting more than 30 small and large joints, discrete patches of hyperpigmentation on his posterior shoulder blades and lower back, erythema overlying multiple knuckles and the dorsal aspect of his distal fingertips, but without associated papules or ulcers (Figure 2), alopecia, and significant weight loss (∼8 kg). At this time, he did not report any neurologic symptoms nor was there any evidence of neurologic dysfunction upon examination. He had associated signs and symptoms of heart palpitations, dyspnea, hair loss, weight loss, dry mouth, and fatigue, without a history of fever, mucocutaneous ulcers, muscle aches and weakness, abdominal pain, constipation, or diarrhea. Labs re-demonstrated mild pancytopenia (WBC, 3.4; hemoglobin, 10.0; platelet count, 122) and evidence of systemic inflammation with an erythrocyte sedimentation rate (ESR) of 43 (reference range, 0–15 mm/h), ferritin of 932 (reference range, 26–388 ng/ml), IgG level of 1,562 (reference range, 550–1,440 mg/dl), and albumin of 3.2 (Table 1A). The C-reactive protein (CRP) was normal. Muscle enzyme markers were elevated, including AST 86 (reference range, 0–35 U/L), alanine transaminase (ALT) of 73 (reference range, 0–50 U/L), aldolase of 14.0 (reference range, 3.3–9.7 U/L), and lactate dehydrogenase (LDH) of 636 (reference range, 0–265 U/L) with normal gamma-glutamyl transferase (GGT) and creatine kinase (CK). Given the elevations of muscle enzymes, a dermatomyositis-associated antibody panel was ordered. Antinuclear antibody (ANA), extractable nuclear antigen (ENA), double-stranded DNA (dsDNA), rheumatoid factor (RF), cyclic citrullinated peptide (CCP), and additional rheumatologic studies were also ordered. Initial imaging results included normal hand radiographs, knee radiographs with small suprapatellar joint effusions, and a normal chest X-ray. A primary rheumatologic etiology, especially SLE or mixed connective tissue disease (MCTD), was suspected in the context of weight loss, alopecia, arthritis, and pancytopenia. We also considered RF-positive polyarticular juvenile idiopathic arthritis as well as early-onset sarcoidosis given the degree of arthritis and dermatitis on presentation. JDM was also suspected in the context of weight loss, arthritis, and fatigue, and this diagnosis became a higher suspicion after the transaminases and LDH returned elevated, as stated above. Systemic infection and malignancy were pursued via additional laboratory testing and an urgent referral to hematology/oncology was placed prior to the initiation of systemic steroids or other immunomodulatory agents. He was started on naproxen 440 mg twice daily.

**Table 1:** Labs at the time of initial evaluation by pediatric rheumatology. (**A**) Initial labs were notable for pancytopenia, systemic inflammation, elevated lactate dehydrogenase (LDH) and aldolase, and transaminitis. (**B**) Additional labs resulting over the following days were notable for a positive antinuclear antibody (ANA) with a negative anti-double-stranded DNA (dsDNA) and extractable nuclear antigen (ENA) antibodies, normal complement levels, normal renal function, and negative infectious workup.

Test	Reference range	Value
**A**		
WBC	4.0–11.0 × 10^3^/ul	3.4
Hemoglobin	11.7–15.7 g/dl	10
Platelet count	150–450 × 10^3^/ul	122
CRP	0.0–8.0 mg/l	<2.9
ESR	0–15	43
Ferritin	26–388	932
Albumin	3.4–5.0 g/dl	3.2
AST	0–35 U/L	86
ALT	0–50 U/L	73
Aldolase	3.3–9.7 U/L	14
LDH	0–265 U/L	636
GGT	0–44 U/L	43
CK	30–300 U/L	126
Uric acid	2.1–6.5 mg/dl	4.1
IgM	26–232 mg/dl	144
IgA	61–348 mg/dl	301
IgG	550–1,440 mg/dl	1,562
**B**		
ANA	<1:40	1:320
dsDNA	<10.0 IU/ml	7.7
ENA panel	Negative	Negative
RF	<12 IU/ml	<6
CCP	<7.0 U/ml	3.4
HLA-B27	Negative	Negative
ACE	18–101 U/L	56
Autoimmune hepatitis panel	Negative	Negative
TSH	0.35–4.94 uIU/ml	4.54
BUN	7–21 mg/dl	13
Creatinine	0.50–1.00 mg/dl	0.43
C3	68–222 mg/dl	95
C4	10–47 mg/dl	26
CH50	38.7–89.9 U/ml	56.5
F-actin	0–19 units	20
Hepatitis B	Non-reactive	Non-reactive
Hepatitis C	Non-reactive	Non-reactive
HIV	Non-reactive	Non-reactive
QuantiFERON Gold	Negative	Indeterminate

ACE, angiotensin converting enzyme; AST, aspartate transferase; BUN, blood urea nitrogen; CCP, cyclic citrullinated peptide; CK, creatine kinase; RF, rheumatoid factor; TSH, thyroid-stimulating hormone; WBC, white blood count; CRP, C-reactive protein; ESR, erythrocyte sedimentation rate; ALT, alanine transaminase; GGT, gamma-glutamyl transferase; HLA-B27, human leukocyte antigen B27.

Over the following days, additional infectious and immunologic testing results were returned (Table 1B). The ANA was found positive (1:320) by the indirect fluorescent antibody test (IFA) with a negative/normal dsDNA antibody, ENA antibody panel, complement studies (C3, C4, CH50), RF, CCP antibody, human leukocyte antigen B27 (HLA-B27), sarcoid screening (angiotensin-converting enzyme and chest X-ray), TSH, and renal testing. An autoimmune hepatitis panel was negative, except for a weakly positive F-actin. The infectious workup was unrevealing, with negative/normal testing for hepatitis B, hepatitis C, and HIV. QuantiFERON Gold returned indeterminate.

**Figure 3:**
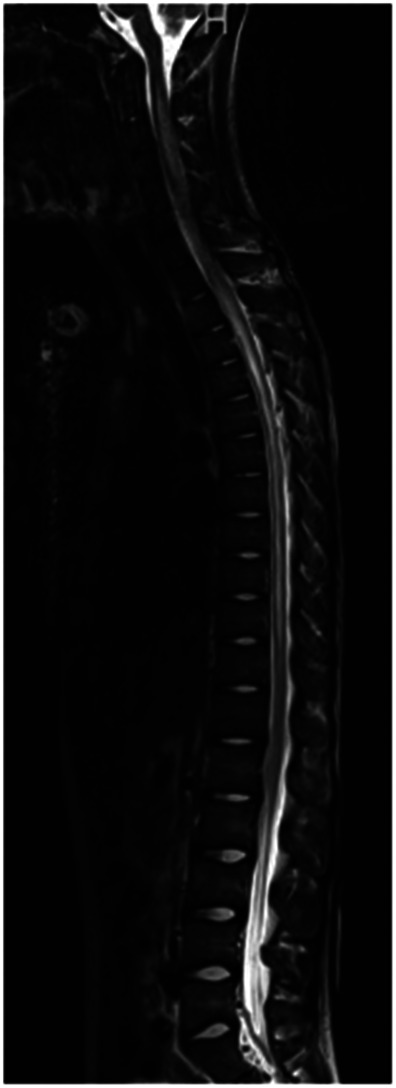
Imaging at the time of presentation to the emergency department. MRI demonstrated extensive T2 signal abnormality involving nearly the entire spinal cord, with hazy enhancement from C5 to T5, as can be seen with transverse myelitis.

Within 1 week of pediatric rheumatology evaluation, the patient developed acute-onset lower extremity weakness, abdominal pain, and inability to urinate, for which he presented to an outside emergency department. A brain and spine MRI was obtained. C5–T5 spinal cord enhancement was observed (Figure 3) concerning for transverse myelitis. The cerebral spinal fluid (CSF) analysis was significant for >2,000 mg/dl protein. Neurology was consulted due to concern for transverse myelitis. The patient underwent extensive workup, including bone marrow biopsy; CT scans of the chest, abdomen, and pelvis; electrocardiogram; and echocardiogram, all of which were normal. The ophthalmology exam was normal; no evidence of optic neuritis was observed. Additional studies of the CSF demonstrated elevated WBC (925 leukocytes/µl), no oligoclonal bands, and mildly elevated IgG. The serum cytokine panel was obtained and was within the reference range. Anti-AQP4 antibody testing assessed by fluorescence-activated cell sorting (FACS) was positive by serum (1:10,000 titer) and CSF (1:2,048 titer), consistent with a diagnosis of NMOSD. The autoimmune CNS evaluation from the serum assessed by IFA, FACS, and cell-based assay (CBA) was otherwise negative, including negative glutamic acid decarboxylase 65 (GAD65) antibodies, myelin oligodendrocyte glycoprotein (MOG) antibodies, and N-methyl-d-aspartate (NMDA-R) antibodies. The paraneoplastic autoantibody panel of the CSF assessed by IFA, FACS, and CBA was otherwise negative, including the anti-NMDA receptor antibody. The full autoimmune CNS panel from the CSF was not completed due to inadequate sampling. The patient was started on daily methylprednisolone infusions (1 g) for 5 days and then transitioned to 60 mg daily prednisone. He received seven therapeutic plasma exchanges and four weekly doses of rituximab at 375 mg/m^2^/dose.

Approximately 3 weeks later, the myositis-associated autoantibody panel returned positive for anti-MDA5 antibodies. His skin findings had also evolved to resemble more classic findings of JDM, including light brown hyperpigmented patches on the bilateral arms, fainter tan to erythematous papules on the bilateral dorsal hands (Gottron papules), and erythematous plaques on the back. He did not have features of the more specific anti-MDA5 cutaneous phenotype, such as palmar papules or ulcerations. Dermatology was consulted, and skin biopsies overlying the left third metacarpophalangeal (MCP) joint and right upper back were performed, which showed features of interface dermatitis. Given the combination of clinical findings (Gottron papules, mechanic hands, and shawl sign), antibody positivity, and skin biopsy findings, he was diagnosed with JDM, consistent with the 2017 European League Against Rheumatism/American College of Rheumatology classification criteria (14). Chest CT did not show signs of ILD. Whole exome sequencing was obtained, and no pathologic variants were identified. He was started on triamcinolone 0.1% ointment for his rashes and transitioned to outpatient rehab.

Following this initial hospitalization, he was discharged to an inpatient rehabilitation service for 2 weeks. In the months following hospitalization, he regained the ability to walk independently, write, and play piano and began regaining weight. His incontinence improved, although he has had ongoing urinary frequency and erectile dysfunction. For remission maintenance therapy, he was started on weekly subcutaneous methotrexate (15 mg/m^2^) and given monthly IVIG (70 g) for both an immunomodulatory effect and for hypogammaglobulinemia that developed following rituximab therapy. In coordination with pediatric neurology, he was additionally re-dosed with rituximab after 6 months, with a plan of continuing it every 6 months for at least a total of 5 years for his NMOSD diagnosis. He has been tapered off of daily steroids. His arthritis resolved, but he had a persistent rash prompting the addition of hydroxychloroquine (200 mg daily).

## Discussion

Here, we present the case of an adolescent patient presenting with anti-MDA5-positive JDM and anti-AQP4 NMOSD. To the best of our knowledge, this is the first described case of anti-MDA5 JDM and NMOSD developing nearly simultaneously in an adolescent patient.

Only two other published cases have described NMOSD with anti-MDA5 antibody positivity and DM. Delman et al. (12) reported the case of a 40-year-old woman who presented with 6 months of fatigue, joint pain, and rash, with exam findings consistent with DM (12). She then developed clinical and radiographic findings diagnostic of transverse myelitis with positive anti-AQP4 antibodies. She also had ILD. She was treated with two courses of high-dose IV steroids, plasma exchange, two infusions of rituximab, and two rounds of IV cyclophosphamide. She was then switched to maintenance therapy with daily prednisone and monthly cyclophosphamide, which was eventually transitioned to azathioprine. She maintained adequate disease control. More recently, Kang et al. (13) described a 35-year-old man who was initially diagnosed with rheumatoid arthritis and ILD after presenting with arthritis and Gottron papules, with labs notable for positive ANA, RF, and CCP and pulmonary fibrosis on chest CT (13). He was started on IV methylprednisolone but developed a sudden elevation in CK and was ultimately diagnosed with DM following a muscle biopsy. Symptoms initially improved on systemic steroids, tacrolimus, and deflazacort, but he later presented to the neurology clinic and was found to have NMOSD with transverse myelitis, optic neuritis, and anti-AQP4 and anti-MDA5 antibody positivity. Following aggressive treatment with high-dose methylprednisolone, tacrolimus, rituximab, and PLEX, he clinically stabilized, but had no improvement in right eye blindness, paraplegia, or bladder dysfunction. Despite striking similarities in these two cases, it is notable that one patient achieved clinical recovery, while the other resulted in significant neurologic disability. Additionally, while one patient developed near-simultaneous symptoms of DM and NMOSD as was the case in our patient, the other had known dermatomyositis, with NMOSD presenting later. These cases also highlight the risk of ILD in MDA5-positive DM (11). Fortunately, at this point, our patient has not had features of ILD.

This case also demonstrates the importance of considering new distinct autoimmune conditions in patients with existing autoimmune diseases and highlights unique management considerations in these circumstances. As seen in our patient, an autoimmune inflammatory myopathy and autoimmune neurologic disease can present nearly simultaneously, and similarities in clinical presentation can make the diagnosis of each disease entity challenging. Proximal muscle weakness or loss of function in these patients could be due to myositis, demyelination, or both. New neurologic disease can also develop in patients with a pre-existing diagnosis of systemic autoimmune disease. Additionally, certain forms of dermatomyositis, including anti-MDA5-associated dermatomyositis, may include amyopathic or only mild muscle disease (11), making prompt diagnosis more challenging. Therefore, clinicians should carefully evaluate new neurologic findings in patients with suspected or known systemic autoimmune disease, recognizing that these findings may represent a separate disease entity. The prompt diagnosis of demyelinating disease has important diagnostic implications as treatment early in the disease course is necessary to minimize tissue damage and prevent or minimize permanent neurologic disability.

The exact mechanisms underlying the pathogenesis of both JDM and NMOSD are incompletely understood. However, identifying the common cellular mediators of these disease entities could offer potential therapeutic targets that can be exploited in severe or refractory disease courses. Both JDM and NMOSD are B-cell mediated and associated with autoantibodies that are hypothesized to be directly pathogenic (2, 11, 15, 16). B-cell-depleting therapies, such as rituximab, are known to be effective at reducing the severity of NMOSD as well as the frequency of relapse (17, 18). Rituximab is not included in current consensus guidelines as a mainstay of treatment in JDM; however, several studies report its use as a potentially efficacious therapy for patients with severe, refractory disease (19–21). Patients with concurrent JDM and NMOSD may therefore benefit from the early introduction of B-cell-depleting therapies, such as rituximab.

Evidence also suggests that hyperactive interferon activation may play a role in the pathogenesis of both JDM and NMOSD, which may be a factor contributing to the near-simultaneous onset of each of these diseases. The upregulation of interferons, particularly type I interferons, has been identified as a typical feature of JDM (11). Anti-MDA5 is a dsRNA receptor that detects viral RNA replication in the cytosol and, upon activation, induces type I interferon expression. In this context, MDA5 autoantibodies may contribute to the dysregulation of IFN activation by direct activation of MDA5 (15). Multiple studies have found that levels of interferon-alpha (IFN-α) are significantly elevated in patients with anti-MDA5 DM, and aberrant activation of interferons has been postulated as a driving factor of rapidly progressive ILD in these patients (22, 23). Elevated IFN activity is also thought to play a role in the pathogenesis of NMOSD, as patients with NMOSD have elevated serum levels of IFN-α that correlates with disease activity (24). This is further supported by multiple reported cases of NMOSD induced by the therapeutic use of recombinant IFN during the treatment of other diseases (25, 26). Therefore, in patients with anti-MDA5 JDM and NMOSD, we hypothesize that there may be a role for the use of IFN blocking therapies, such as JAK inhibitors, to more directly target the IFN hyperactivity that occurs in both disease processes. JAK inhibitors have not been used in our patient, and further studies are needed to determine whether they could be an effective therapy for patients with anti-MDA5 JDM and NMOSD.

In summary, adolescent patients can develop a near-simultaneous onset of anti-MDA5-positive JDM and anti-AQP4 NMOSD, and the prompt diagnosis and treatment of these conditions are critical to prevent permanent disability. These patients may benefit from the early introduction of B-cell-targeting therapies or potential JAK inhibitors for refractory disease.

## Data Availability

The original contributions presented in the study are included in the article/Supplementary Material, further inquiries can be directed to the corresponding author.
